# Broad Lipidomic and Transcriptional Changes of Prophylactic PEA Administration in Adult Mice

**DOI:** 10.3389/fnins.2019.00527

**Published:** 2019-06-11

**Authors:** Raissa Lerner, Diego Pascual Cuadrado, Julia M. Post, Beat Lutz, Laura Bindila

**Affiliations:** Institute of Physiological Chemistry, University Medical Center of the Johannes Gutenberg University Mainz, Mainz, Germany

**Keywords:** PEA, PUFAs, inflammation, targeted lipidomics, phospholipids, endocannabinoids, mRNA

## Abstract

Beside diverse therapeutic properties of palmitoylethanolamide (PEA) including: neuroprotection, inflammation and pain alleviation, prophylactic effects have also been reported in animal models of infections, inflammation, and neurological diseases. The availability of PEA as (ultra)micronized nutraceutical formulations with reportedly no side effects, renders it accordingly an appealing candidate in human preventive care, such as in population at high risk of disease development or for healthy aging. PEA’s mode of action is multi-facetted. Consensus exists that PEA’s effects are primarily modulated by the peroxisome proliferator-activated receptor alpha (PPARα) and that PEA-activated PPARα has a pleiotropic effect on lipid metabolism, inflammation gene networks, and host defense mechanisms. Yet, an exhaustive view of how the prophylactic PEA administration changes the lipid signaling in brain and periphery, thereby eliciting a beneficial response to various negative stimuli remains still elusive. We therefore, undertook a broad lipidomic and transcriptomic study in brain and spleen of adult mice to unravel the positive molecular phenotype rendered by prophylactic PEA. We applied a tissue lipidomic and transcriptomic approach based on simultaneous extraction and subsequent targeted liquid chromatography-multiple reaction monitoring (LC-MRM) and mRNA analysis by qPCR, respectively. We targeted lipids of COX-, LOX- and CYP450 pathways, respectively, membrane phospholipids, lipid products of cPLA_2_, and free fatty acids, along with various genes involved in their biosynthesis and function. Additionally, plasma lipidomics was applied to reveal circulatory consequences and/or reflection of PEA’s action. We found broad, distinct, and several previously unknown tissue transcriptional regulations of inflammatory pathways. In hippocampus also a PEA-induced transcriptional regulation of neuronal activity and excitability was evidenced. A massive downregulation of membrane lipid levels in the splenic tissue of the immune system with a consequent shift towards pro-resolving lipid environment was also detected. Plasma lipid pattern reflected to a large extent the hippocampal and splenic lipidome changes, highlighting the value of plasma lipidomics to monitor effects of nutraceutical PEA administration. Altogether, these findings contribute new insights into PEA’s molecular mechanism and helps answering the questions, how PEA prepares the body for insults and what are the “good lipids” that underlie this action.

## Introduction

Since diet is an essential environmental factor to maintain health, it is important to advance research in the field of nutrition science and nutraceutical use in order to expedite disease prevention and/or hinder disease progression. Thus, we need more holistic biology approaches for nutrition research, including multi-omic strategies to investigate body’s response to nutrition, and to validate and understand its beneficial effects (Allison et al., 2015). An advantage of nutrition-based approaches over, or in addition to, pharmacological ones for disease prevention and treatment is the opportunity for extended administration periods, due to the reduced number of possible side-effects. Palmitoylethanolamide (PEA), a saturated fatty acid (16:0) of the *N*-acylethanolamines family, is a food component found in egg yolk, soybeans and peanuts and was investigated for more than half a century and described in numerous studies to exert anti-inflammatory effects (Lambert et al., 2001; Conti et al., 2002; Keppel Hesselink et al., 2013; Mattace Raso et al., 2014; Rinne et al., 2018). PEA is also a natural own body compound found in most cell types, tissues, and bodily fluids. It is synthesized and metabolized via different enzymes, namely *N*-acyl phosphatidylethanolamine phospholipase D (NAPE-PLD), fatty acid amid hydrolase (FAAH) and/or *N*-acylethanolamine acid amidase (NAAA), thus sharing the same biosynthetic pathway with the endocannabinoid (eCB) anandamide (AEA) (Petrosino and Di Marzo, 2017). Several clinical trials proved that exogenous administration of PEA lacks side effects, and since 2008 it has been marketed in different countries as a nutraceutical food supplement (Keppel Hesselink et al., 2013; Artukoglu et al., 2017). Despite many clinical trials and a number of papers describing the therapeutic role of PEA in chronic pain, inflammation and neurodegenerative diseases, its mechanism of action is not yet clarified (Iannotti et al., 2016). PEA was evidenced to act through receptor binding, and was initially thought to bind to the cannabinoid 2 receptor (CB2) (Facci et al., 1995). Further research however, revealed that PEA, unlike AEA, exhibits only weak binding efficacy on the CB2 receptor, but possesses the capability to affect AEA signaling by acting as a competing substrate (Di Marzo et al., 2001). In recent years compelling evidence accumulated on the peroxisome proliferator-activated receptor α (PPARα) as the main molecular target of PEA (LoVerme et al., 2006; Hansen, 2010). PPARα is an ubiquitous transcription factor exerting a major role in lipid metabolism, e.g., was recently described to protect against diet-induced obesity in mice (Araki et al., 2018). Gene networks regulated by PPARα-PEA signaling lead to the reduction of the transcription of pro-inflammatory genes (Lo Verme et al., 2005; D’Agostino et al., 2007, 2009; Hansen, 2010). Another described direct target of PEA is the orphan receptor GPR55 (Ryberg et al., 2007). PEA’s actions were also attributed to effects upon ATP-sensitive K^+^-channels (Romero and Duarte, 2012), TRP channels (Lowin et al., 2015), and NFkB (D’Agostino et al., 2009). The role of lipid amides including of PEA, on down-modulation of mast cell activation has been demonstrated in the seminal work of Nobel prize laureate Rita Levi-Montalcini (Aloe et al., 1993), and has since prompted a body of work evidencing the mast cells as targets for the various anti-inflammatory effects of PEA (Skaper, 2017). However, the various molecular mechanisms underlying the multi-facetted effects of PEA, e.g., neuroprotective, anti-inflammatory, anticonvulsant, pain killer warrant further investigation, especially considering the emerging interest into its use as a prophylactic and therapeutic adjuvant.

We recently demonstrated neuroprotective, anticonvulsant and anti-inflammatory effects of sub- chronic, prophylactic PEA administration in a mouse model for acute epileptic seizures and found that these effects were accompanied by alterations in peripheral and hippocampal eCB and prostaglandin levels (Post et al., 2018). Similarly, prophylactic PEA improved survival and decreased inflammation in mice models with bacterial meningitis and these effects were in part accompanied by eicosanoids (eiCs) modulation (Heide et al., 2018).

In this study, we aim to expand our understanding of how the PEA, as a saturated lipid with a relatively long fatty acyl chain (C16:0), alters the lipid tissue composition, metabolism and signaling upon prophylactic administration such that it actually positively influence the immune system, neuroprotective functions and improve symptomatic when the body faces various insults (neuroinjury, infections, etc.). For this purpose, we applied a recently developed tissue lipidomics and transcriptomic approach (Lerner et al., 2018) to investigate changes of multiple lipid categories, e.g., eCBs, eiCs, poly unsaturated fatty acids (PUFAs), PUFA oxidation products, phospholipids (PLs), and different sphingosine species, as well as related genes involved in lipid signaling and metabolism in hippocampus (HC) and spleen of control mice. The choice of hippocampus was guided by its known role in the on-set of neurological diseases including epilepsy (Heide et al., 2018; Lerner et al., 2018), and the previously demonstrated effect of PEA to alleviate hippocampal neuroinflammation and endocannabinoids elevation (Post et al., 2018) at acute seizure state. The spleen has been increasingly recognized to have a unique function in immune responses, including clearance of cell debris and antigens from the blood stream, as well as an early host response to infections (Mebius and Kraal, 2005; Wluka and Olszewski, 2006; Bronte and Pittet, 2013). As the body’s most proximal and largest blood filtering organ, with ability to mount innate and adaptive immune responses, we rationalized that the prophylactic and particularly the immunomodulatory effects of PEA (Lowin et al., 2015; Skaper, 2017; Post et al., 2018) could be partly attributed to eliciting splenic molecular changes. Hence, targeted lipidomics and transcriptomics were applied on spleen of adult (wild type) mice with and without PEA administration. In addition, circulatory levels of the target lipids were assessed for their value to reflect brain and peripheral molecular effects of prophylactic PEA. To our knowledge, most studies on PEA’s action focus on gene networks and inflammatory mediators, whereas no broad lipidomic studies are reported so far. Hence, this study is the first to give a more thorough insight into the transcriptome and lipidome plasticity underlying the prophylactic and nutritional benefits of PEA.

## Materials and Methods

### Reagents and Chemicals

Calibration Standards: arachidonoyl ethanolamide (AEA), 2-arachidonoyl glycerol (2-AG), arachidonic acid (AA), resolvin D1 (RvD1), prostaglandin D2 (PGD_2_), prostaglandin E2 (PGE_2_), 11-beta-prostaglandin F2alpha (11β-PGF_2_α), 5S-hydroxyeicosatetraenoic acid (5(S)-HETE), 12(S)-hydroxyeicosatetraenoic acid (12(S)-HETE), 15(S)-hydroxyeicosatetraenoic acid (15(S)-HETE), and 20-hydroxyeicosatetraenoic acid (20-HETE) were obtained from BIOMOL Research Laboratories, Inc. (Plymouth Meeting, PA, United States). Linoleic acid (LA), alpha-linolenic acid (ALA), docosahexaenoic acid (DHA), docosapentaenoic acid (DPA), and eicosapentaenoic acid (EPA) were purchased from Cayman Chemical (Ann Arbor, MI, United States). Phosphatidylcholine 16:0/18:1 (PC 16:0/18:1), phosphatidylglycerol 16:0/18:1 (PG 16:0/18:1), phosphatidylethanolamine 16:0/18:1 (PE 16:0/18:1), phosphatidylserine 16:0/18:1 (PS 16:0/18:1), phosphatidic acid 16:0/18:1 (PA 16:0/18:1), phosphatidylinositol 16:0/18:1 (PI 16:0/18:1), lysophosphatidylcholine 18:0/0:0 (LPC 18:0), lysophosphatidic acid 16:0/0:0 (LPA 16:0), sphingomyelin d18:1/18:0 (SM d18:1/18:0), ceramide-1-phosphate d18:1/16:0 (C1P d18:1/16:0), sphingosine d18:1 (SPH d18:1) and sphingosine-1-phosphate d18:1 (S1P d18:1) were obtained from Avanti Polar Lipids, Inc. (Alabaster, AL, United States).

Internal standards (ISTDs) for quantification: arachidonoyl ethanolamide-d_4_ (AEA-d_4_), 2-arachidonoyl glycerol-d_5_ (2-AG-d_5_), arachidonic acid-d_8_ (AA-d_8_), prostaglandin D2-d_4_ (PGD_2_-d_4_), prostaglandin E2-d_9_ (PGE_2_-d_9_), 5(S)-hydroxyeicosatetraenoic acid-d_8_ (5(S)-HETE-d_8_), 12(S) hydroxyeicosatetraenoic acid-d_8_ (12(S)-HETE-d_8_), 20-hydroxyeicosatetraenoic acid-d_6_ and resolvin D1-d_5_ (RvD1-d_5_) were obtained from BIOMOL Research Laboratories, Inc. (Plymouth Meeting, PA, United States). Linoleic acid-d_4_ (LA-d_4_), alpha-linolenic acid-d_5_ (ALA-d_5_), docosahexaenoic acid-d_5_ (DHA-d_5_), docosapentaenoic acid-d_5_ (DPA-d_5_) and eicosapentaenoic acid-d_5_ (EPA-d_5_) were purchased from Cayman Chemical (Ann Arbor, MI, United States). Phosphatidylcholine 17:0/14:1 (PC 17:0/14:1), phosphatidylglycerol 17:0/14:1 (PG 17:0/14:1), phosphatidylethanolamine 17:0/14:1 (PE 17:0/14:1), phosphatidylserine 17:0/14:1 (PS 17:0/14:1), phosphatidic acid 17:0/14:1 (PA 17:0/14:1), phosphatidylinositol 17:0/14:1 (PI 17:0/14:1), lysophosphatidylcholine 17:0/0:0 (LPC 17:0), lysophosphatidic acid 17:0/0:0 (LPA 17:0), sphingomyelin d18:1/12:0 (SM d18:1/12:0), ceramide-1-phosphate d18:1/12:0 (C1P d18:1/12:0), and sphingosine d17:1 (SPH d17:1), sphingosine-1-phosphate d17:1 (S1P d17:1) were obtained from Avanti Polar Lipids, Inc. (Alabaster, AL, United States).

Water, *n*-hexane, ethylacetate, methanol, 2-propanol, acetonitrile, chloroform, formic acid, and ammonium formate of liquid chromatography/mass spectrometry (LC/MS) grade were invariably used (Sigma-Aldrich, St. Louis, MO, United States) for extraction and LC/multiple reaction monitoring (MRM) analysis. HPLC-grade methyl tert-butyl ether (MTBE), Trizma^®^ hydrochloride solution (Tris-HCl) (pH 7.4), triethylamine (TEA) and butylhydroxytoluene (BHT) were purchased from Sigma-Aldrich (St. Louis, MO, United States). URB597 (KDS-4103, 3′-(aminocarbonyl) [1, 1′-biphenyl]-3-yl -cyclohexylcarbamate) was purchased from Cayman Chemical (Ann Arbor, MI, United States), tetrahydrolipstatin (THL) was obtained from Santa Cruz Biotechnology, Inc. (Dallas, TX, United States), and β-mercaptoethanol was obtained from Carl Roth (Karlsruhe, Germany).

### Animals and PEA Administration

**FIGURE 1:**
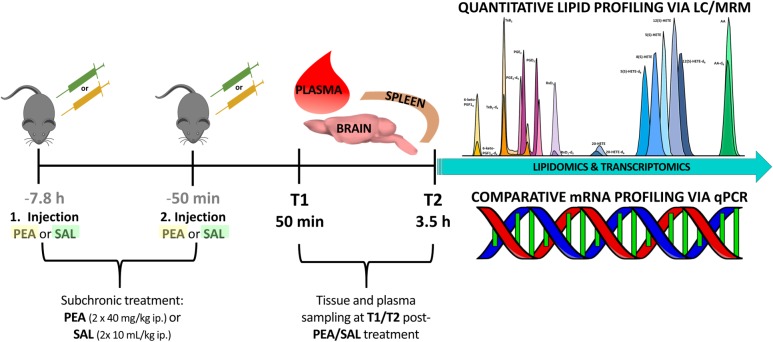
Experimental timeline of PEA/Sal treatment: in total 24 mice were ip injected either with saline (*n* = 12) or with a dose of 40 mg/kg PEA (*n* = 12) each in a total volume of 10 mL/kg **(1^st^ injection, at 7.8 h prior to sacrificing)**. After 7 h the same procedure was repeated **(2^nd^ injection, at 50 min prior to sacrificing)**. At **time point 1** (50 min post – 2^nd^ injection) 12 mice were sacrificed and plasma, brain, and spleen samples from PEA-treated (*n* = 6) and saline-treated (*n* = 6) mice were collected for further analysis. This procedure was repeated with remaining 12 mice, e.g., PEA-treated (*n* = 6) and saline-treated (*n* = 6) mice, at **time point 2** (3.5 h post – 2^nd^ injection).

Experiments were performed according to the European Community’s Council Directive of 22 September 2010 (2010/63EU) and approved by the local animal care committee of the German Rhineland-Palatinate (file reference: 23 177-07/G16- 1-075). Experimental procedures were conducted using 8–10 weeks old C57BL/6N male mice obtained from Janvier Labs (Saint-Berthevin, France). Mice (*n* = 24) were group housed (3–4/cage) on a 12-h light/dark schedule under environmental conditions (20–22°C and 55–60% humidity) with water and food available *ad libitum* for at least 1 week prior to commencement of experiments. In order to unravel the benefits of prophylactic PEA administration and its impact on lipidome and transcriptome level, mice were sub-chronically PEA-treated (*n* = 12) at two time points, at 7.8 h and at 50 min prior to sacrificing, via intraperitoneal (ip) injection with a dose of 40 mg/kg in 10 mL/kg, respectively. PEA was freshly dissolved in DMSO/chremophore/saline (17:2:1) and kept in thermo-mixer (low speed) at 33°C until injection to avoid precipitation. Control mice (*n* = 12) were ip injected with 0.9% saline in 10 mL/kg. Mice treated with PEA or saline (Sal) 7 h prior to 2^nd^ injection, respectively. Six mice per group (PEA/Sal) were sacrificed 50 min after the 2^nd^ injection (time point 1) and 3.5 h after 2^nd^ -injection (time point 2), respectively (Figure 1). The animal experiments were carried out by one trained scientist. Visual assessment of animal behavior upon PEA administration indicated no noticeable changes.

### Spleen, Hippocampus, and Plasma Sampling

At each of the two time points 12 mice were shortly anesthetized with isoflurane and sacrificed by decapitation. Brains and spleens were isolated and immediately frozen on dry ice. Brain dissection was carried out according to the protocol previously described (Lerner et al., 2016). Spleens were stored at -80°C and pieces of 25–30 mg per spleen were cut and used for extraction and further analysis. Plasma sampling and handling prior to extraction was performed as described previously (Post et al., 2018).

### Simultaneous RNA and Lipid Extraction From Spleen and Hippocampus

Simultaneous extraction of RNA and lipids from spleen and HC was conducted via the protocol established in Lerner et al. (2018). Briefly, 200 μL chloroform, ice-cold ceramic beads and 600 μL of RLT buffer (supplied with the RNeasy^®^ Mini Kit) containing 1% β-mercaptoethanol, 5 μM THL/URB597 and 10 μg/mL BHT in final volume were added to the frozen tissue samples, obtained from the PEA- and Sal injected animals (*n* = 12 for each group). The aliquots were spiked with 10 μl ISTDs to a target concentration of 150 ng/mL PC 17:0/14:1; PE 17:0/14:1; PA 17:0/14:1, 100 ng/mL PG 17:0/14:1; PS 17:0/14:1; PI 17:0/14:1; LPC 17:0; LPA 17:0; SM d18:1/12:0; EPA-d_5_; DPA-d_5_; DHA-d_5_; 2-AG-d_5_, 1000 ng/ml LA-d_4_; ALA-d_5_, 500 ng/ml AA-d_8_; C1P d18:1/12:0, 200 ng/ml SPH d17:1; S1P d17:1 and 0.5 ng/ml AEA-d_4_ respectively, in the final volume. After homogenization via Precellys 24 (Peqlab, Erlangen, Germany) (6000 rpm; 20 s) and subsequent centrifugation for 5 min at full speed and 4°C, the upper phase was used for further RNA extraction via the RNeasy^®^ Mini Kit according to the manufacturer’s instructions (RNase-Free DNase Set, Qiagen, Hilden, Germany) and the lower chloroform-containing phase was further used for lipid extraction (Lerner et al., 2018).

In our study, we used a previously established protocol for lipidomic and transcriptomic profiling, due to its amenability for simultaneous lipid and RNA extraction from the same tissue sample, and pertaining the imperative standard operating procedure in regard to tissue/plasma sampling, sample handling and storage conditions, in order to reduce artificial analyte alterations (Lerner et al., 2016, 2018). Quantification of the lipid analytes via LC/MRM with on-line polarity switching enabled investigation of several lipid species encompassing 9 PL classes as well as eCBs in single experiments, respectively. In order to attain a broader view of lipid plasticity related to PEA’s action, we adapted the MRM parameters to allow additional analysis of PUFAs and additional sphingolipid species (Table 1).

**Table 1:** Targeted ion transitions.

Positive ion mode					
**Calibration standards and quantified PLs**			**Corresponding internal standards**		
	
**Analyte Name**	**Precursor ion *m/z***	**Product ion *m/z***	**Analyte name**	**Precursor ion *m/z***	**Product ion *m/z***

2-AG	379.1	287.2	2-AG-d_5_	384.2	287.2
AEA	348.3	62.1	AEA-d_4_	352.3	66.1
LPC 18:0	524.37	184.07	LPC 17:0	510.36	184.07
LPC 20:4	544.34	184.07			
PC 16:0/18:1	760.59	184.07	PC 17:0/14:1	718.54	184.07
PC 38:6	806.67	184.07			
PC 38:4	810.66	184.07			
SM d18:1/18:0	731.61	184.07	SM d18:1/12:0	647.51	184.07
SM 34:1	703.57	184.07			
SPH d18:1	300.28	282.2	SPH d17:1	286.47	268.2
**Negative ion mode**					

**Calibration standards and quantified PLs**			**Corresponding internal standards**		
	
**Analyte Name**	**Precursor ion *m/z***	**Product ion *m/z***	**Analyte name**	**Precursor ion *m/z***	**Product ion *m/z***

AA	303.05	259.1	AA-d_8_	311.04	267.0
LA	279.23	261.1	LPA 17:0	423.25	153.00
ALA	277.22	259.1	ALA-d_ 5_	282.2	238.1
GLA	277.22	259.1			
EPA	301.22	257.2	EPA-d_5_	306.25	262.2
18-HEPE	317.22	259.0			
DPA	329.25	285.2	DPA-d_ 5_	334.3	290.2
DHA	327.23	283.2	DHA-d_ 5_	332.26	288.2
17(S)-HDHA	343.24	201.0			
LPA 16:0	409.24	153.00	LPA 17:0	423.25	153.00
LPA 20:4	457.24	153.00			
PA 16:0/18:1	673.48	255.23	PA 17:0/14:1	631.43	269.25
PS 16:0/18:1	760.51	255.23	PS 17:0/14:1	718.47	269.25
PS 36:4	782.49	303.23			
PS 38:4	810.53	303.23			
PI 16:0/18:1	835.53	281.25	PI 17:0/14:1	793.49	269.25
PI 36:4	857.52	303.23			
PI 38:4	885.55	303.23			
LPI 20:4	619.29	303.23			
PG 16:0/18:1	747.52	281.25	PG 17:0/14:1	705.47	225.19
PG 36:5	767.49	303.23			
PG 38:5	795.52	303.23			
PE 16:0/18:1	716.52	281.25	PE 17:0/14:1	674.48	225.19
PE 38:6	762.51	303.23			
PE 38:4	766.54	303.23			
PE 40:6	790.54	303.23			
PE 40:4	794.57	303.23			
C1P d18:1/16:0	616.47	78.9	C1P d18:1/12:0	560.41	78.9
S1P d18:1	378.24	78.9	S1P d17:1	364.23	78.9
5(S)-HETE	319.23	115.0	5(S)-HETE-d_8_	327.23	116.0
12(S)-HETE	319.23	179.0	12(S)-HETE-d_8_	327.23	184.0
15(S)-HETE	319.23	219.0	5(S)-HETE-d_8_	327.23	116.0
20-HETE	319.23	289.0	20-HETE-d_6_	325.23	295.0
PGE_2_	351.23	315.3	PGE_2_-d_9_	360.25	351.23
PGD_2_	351.23	315.3	PGD_2_-d_4_	355.25	275.3
RvD1	375.22	215.1	RvD_1_-d_5_	380.22	180.2
11β-PGF_2_α	353.24	193.0	PGD_2_-d_4_	355.25	275.3


An additional lipid extraction was carried out from the same tissue origin (see above for PLs and eCBs) followed by the LC/MRM analysis to target lipids of the COX- LOX and CYP450 pathways namely RvD1, HETEs, PGD_2_, 11β-PGF_2_α and PGE_2_. Analysis of LOX/CYP450/COX-derived lipids was carried out in this case in negative ion mode (Post et al., 2018).

**FIGURE 2:**
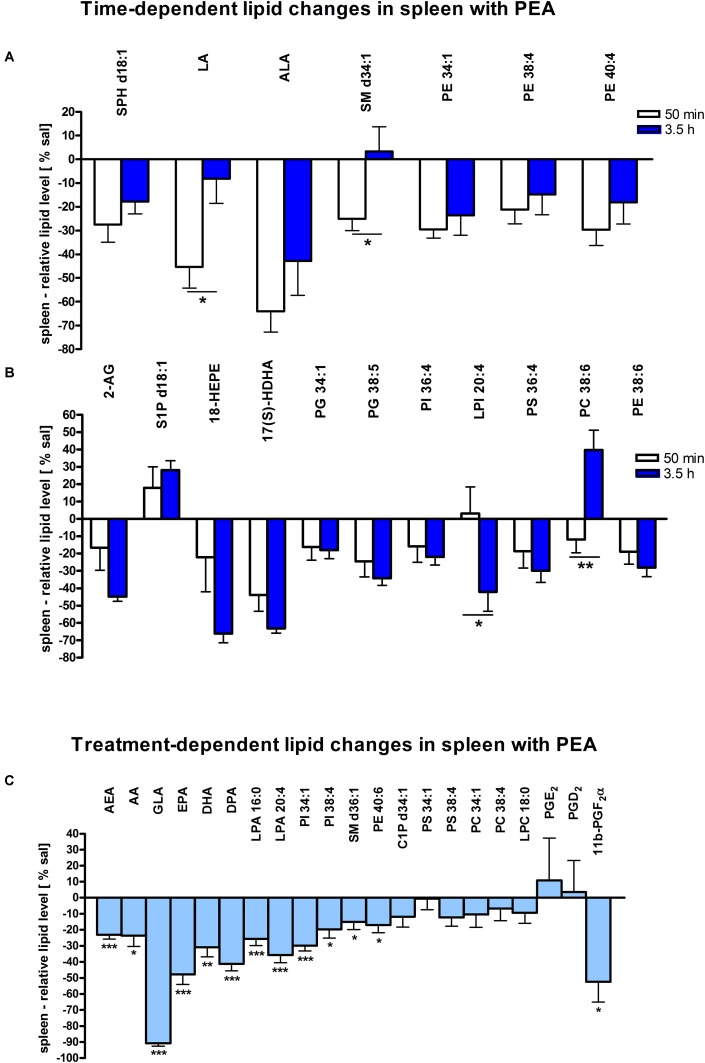
Lipid levels of spleen after PEA treatment: relative lipid concentrations [normalized to the tissue weight (spleen, approximately 20 mg)] after PEA treatment as percentage of saline treated mice are shown and presented as mean value ± SEM. **(A) Time point 1:** time-specific lipid plasticity after 50 min of PEA treatment. Relative lipid levels for the two different time points are shown for reference, but only lipids significantly changing after 50 min of PEA treatment are depicted. **(B) Time point 2:** time specific lipid plasticity after 3.5 h of sub chronic PEA treatment. Relative lipid level for two different time points are shown and only lipids significantly changing after 3.5 h of PEA treatment are depicted. Significant differences between the time points were also detected and as they underline time specificity for these lipids, they were marked on the figure with ^∗^. **(C) Merged time points:** PEA treatment effect on lipid plasticity. Changes for AA, PI 38:4, SM d36:1, PE 40:6 and 11β-PGF_2_α are significant when pooling the time points. The remaining significantly changed lipids show significant changes for both time points, respectively. Data were considered significant at a *p*-value < 0.05, e.g., ^∗∗∗^*p* < 0.001, ^∗∗^*p* = 0.001 to 0.01; ^∗^*p* = 0.01 to 0.05.

Assessment of lipid/transcriptional analyte plasticity in spleen, plasma and brain was conducted to unravel interrelations between brain and periphery after PEA injection. The obtained values of lipid levels from spleen and plasma are normalized to saline values and presented as percentage and depicted in Figure 2, 3 as obtained after 50 min of PEA injection, 3.5 h and pooled time points, respectively. The changes for LPC 20:4, which was the only lipid to be changed in every investigated region, are shown in Figure 4. Changes for HETEs are depicted in Figure 5. Splenic concentrations of RvD1 after PEA administration are shown in Figure 6. The changes in mRNA levels for spleen and HC for the different time points are shown in Figure 7, 8. A simplified signaling scheme for all investigated analytes is depicted in Figure 9.

**FIGURE 3:**
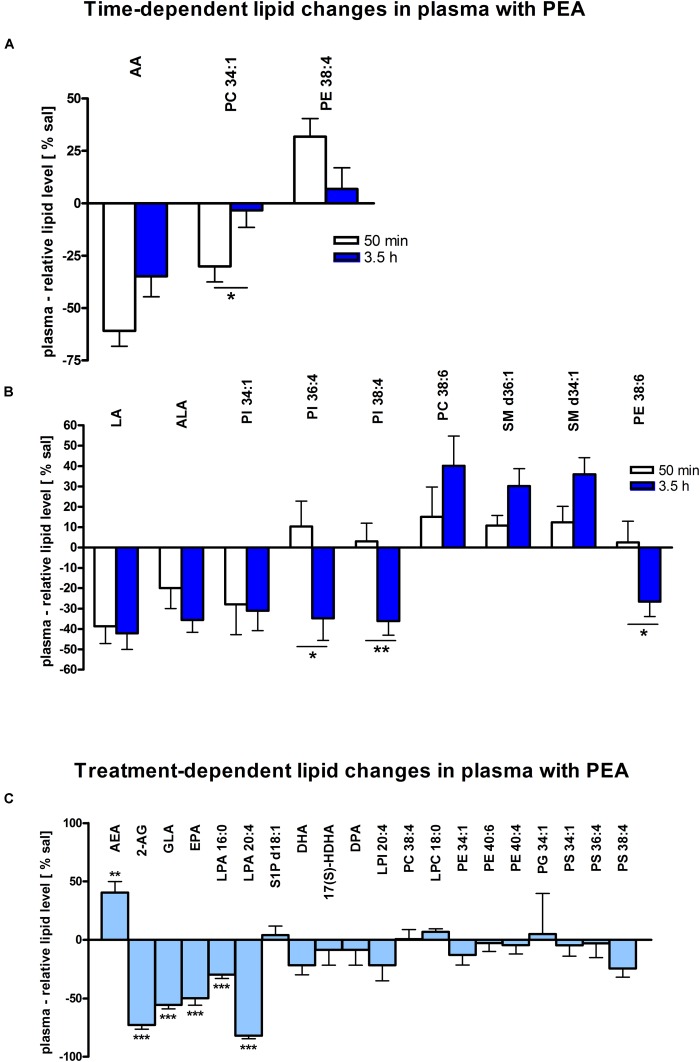
Lipid levels of plasma after PEA treatment. Relative lipid concentrations (normalized to ml plasma: 40 μl) after PEA treatment as percentage of saline treated mice are shown and presented as mean value ± SEM. **(A) Time point 1:** time-specific lipid plasticity after 50 min of PEA treatment. Relative lipid levels for two different time points are shown and only lipids significantly changing after 50 min of PEA treatment are depicted. **(B) Time point 2:** time specific lipid plasticity after 3.5 h of sub-chronic PEA treatment. Relative lipid level for two different time points are shown and only lipids significantly changing after 3.5 h of PEA treatment are depicted. Significant differences between the time points were also detected and as they underline time specificity for these lipids, they were marked on the figure with ^∗^. **(C) Merged time points:** PEA treatment effect on lipid plasticity. Changes for AA, PI 38:4, SM d36:1 and PE 40:6 are significant when pooling the time points. The remaining significantly changed lipids show significant changes for both time points, respectively. Data were considered significant at a *p*-value < 0.05, e.g., ^∗∗∗^*p* < 0.001, ^∗∗^*p* = 0.001 to 0.01; ^∗^*p* = 0.01 to 0.05.

**FIGURE 4:**
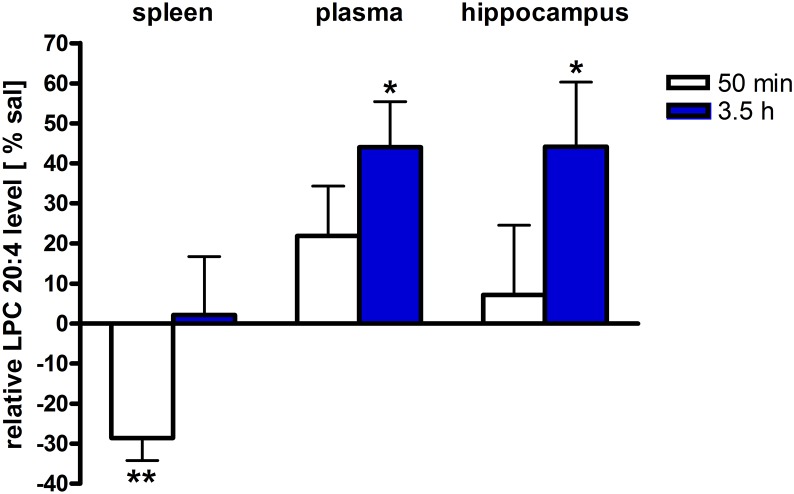
LPC 20:4 levels of brain and periphery after PEA treatment. Relative LPC 20:4 concentrations (normalized to ml plasma/tissue weight) after PEA treatment as percentage of saline treated mice are shown and presented as mean value ± SEM. Relative lipid level for two different time points are depicted. Time specific changes in LPC 20:4 levels occur in spleen at 50 min while in plasma and hippocampus at 3.5 h after PEA treatment. Data were considered significant at a *p*-value < 0.05, e.g., ^∗∗∗^*p* < 0.001, ^∗∗^*p* = 0.001 to 0.01; ^∗^*p* = 0.01 to 0.05.

**FIGURE 5:**
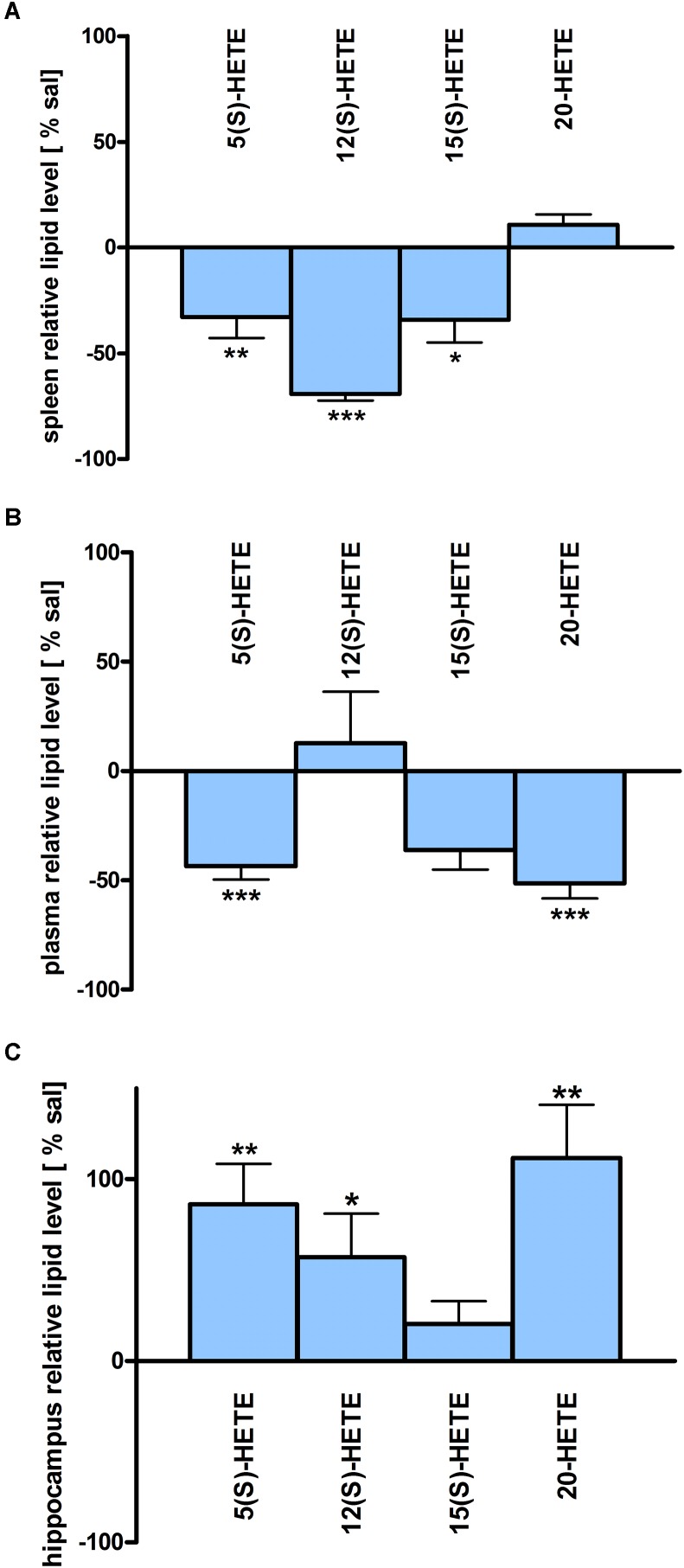
HETE lipid levels in brain and periphery after PEA treatment: relative HETE concentrations (normalized to ml plasma/tissue weight) after PEA treatment as percentage of saline treated mice are shown and presented as mean value ± SEM. **(A) Spleen:** HETE alterations with sub-chronic PEA treatment and unchanged HETE levels in spleen. Only significant reductions of HETE levels can be found in spleen. **(B) Plasma** HETE alterations with sub-chronic PEA treatment and unchanged HETE levels in plasma. Only significant reductions of HETE levels can be found in plasma. **(C) Hippocampus:** HETE alterations with sub chronic PEA treatment and unchanged HETE levels in hippocampus. Only significant increments of HETE levels can be found in hippocampus. Data were considered significant at a *p*-value < 0.05, e.g., ^∗∗∗^*p* < 0.001, ^∗∗^*p* = 0.001 to 0.01; ^∗^*p* = 0.01 to 0.05.

**FIGURE 6:**
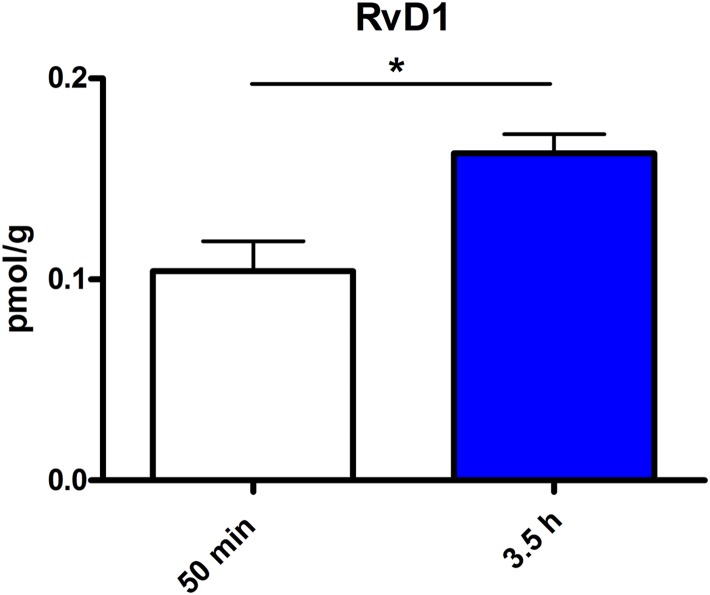
RvD1 concentration in spleen after different time points of PEA treatment: absolute concentrations of RvD1 [normalized to the tissue weight (spleen ≙ 20 mg)] after two time points of PEA treatment presented as mean value ± SEM. RvD1 is significantly increased after 3.5 h of PEA treatment as compared to 50 min. Of note, RvD1 was not detected in saline-treated control mice, likely because they are at basal level under limit of detection/quantification or not biosynthesized. Data were considered significant at a *p*-value < 0.05, e.g., ^∗∗∗^*p* < 0.001, ^∗∗^*p* = 0.001 to 0.01; ^∗^*p* = 0.01 to 0.05.

**FIGURE 7:**
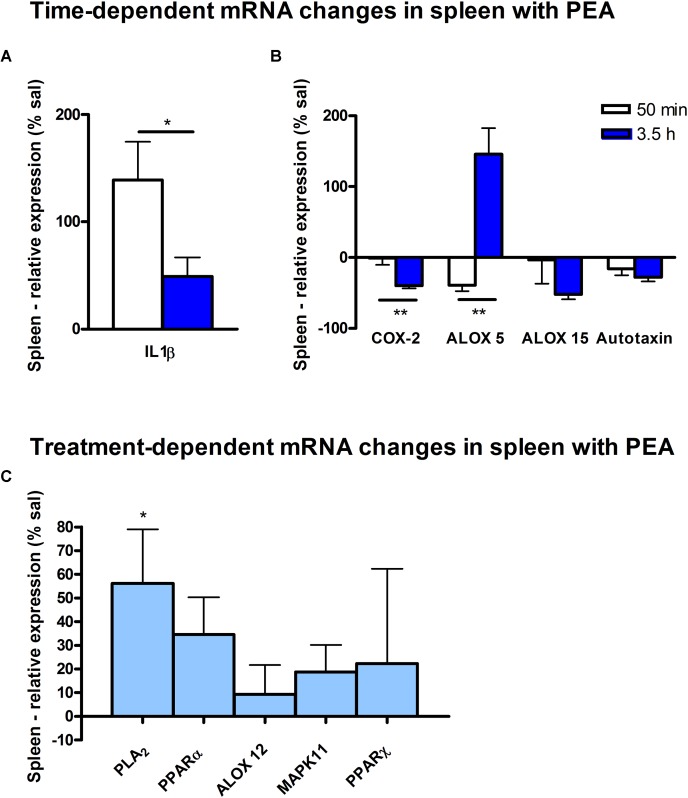
mRNA levels of spleen after PEA treatment: relative mRNA values after PEA treatment as percentage of saline-treated mice, presented as mean value ± SEM. **(A) Time point 1:** time specific mRNA changes after 50 min of sub-chronic PEA treatment. Relative mRNA levels for two different time points are shown and only genes significantly changing after 50 min of PEA treatment are depicted. **(B) Time point 2:** time-specific mRNA changes after 3.5 h of PEA treatment. Relative mRNA levels for two different time points are shown and only genes significantly changing after 3.5 h of PEA treatment are depicted. Significant differences between the time points underline time specificity for these mRNAs and are depicted on the graph when occurring. **(C) Merged time points:** relative mRNA levels for merged time points. Changes for cPLA_2_ are significant when pooling the time points. Data were considered significant at a *p*-value < 0.05, e.g., ^∗∗∗^*p* < 0.001, ^∗∗^*p* = 0.001 to 0.01; ^∗^*p* = 0.01 to 0.05.

**FIGURE 8:**
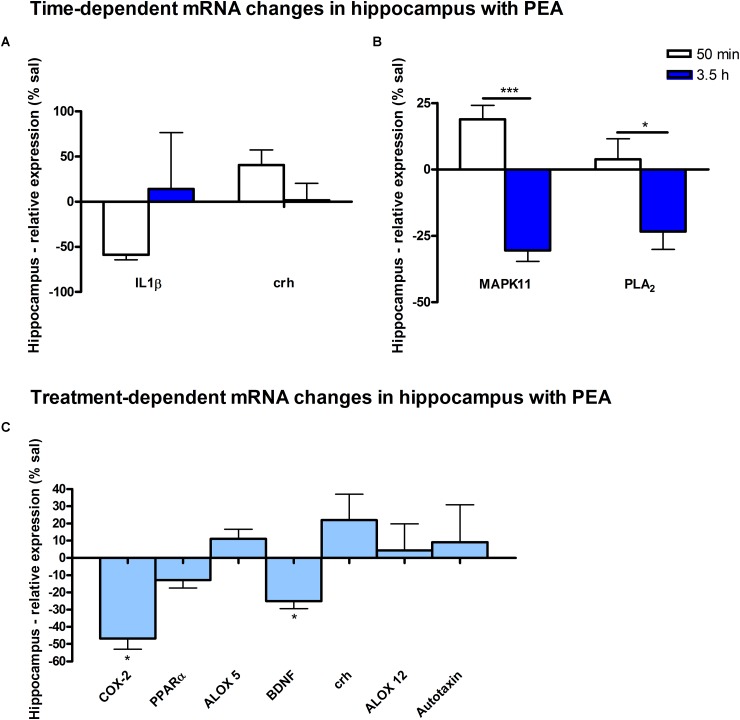
mRNA levels of hippocampus after PEA treatment: relative mRNA values after PEA treatment as percentage of saline treated mice, presented as mean value ± SEM. **(A) Time point 1:** time specific mRNA changes after 50 min of sub-chronic PEA treatment. Relative mRNA levels for two different time points are shown and only genes significantly changing after 50 min of PEA treatment are depicted. **(B) Time point 2:** time-specific mRNA changes after 3.5 h of PEA treatment. Relative mRNA levels for two different time points are shown and only genes significantly changing after 3.5 h of PEA treatment are depicted. Significant differences between the time points underline time specificity for these mRNAs and are depicted on the graph when occurring. **(C) Merged time points:** relative mRNA levels for merged time points. Changes for COX-2 and Bdnf are significant when pooling the time points. Data were considered significant at a *p*-value < 0.05, e.g., ^∗∗∗^*p* < 0.001, ^∗∗^*p* = 0.001 to 0.01; ^∗^*p* = 0.01 to 0.05.

**FIGURE 9:**
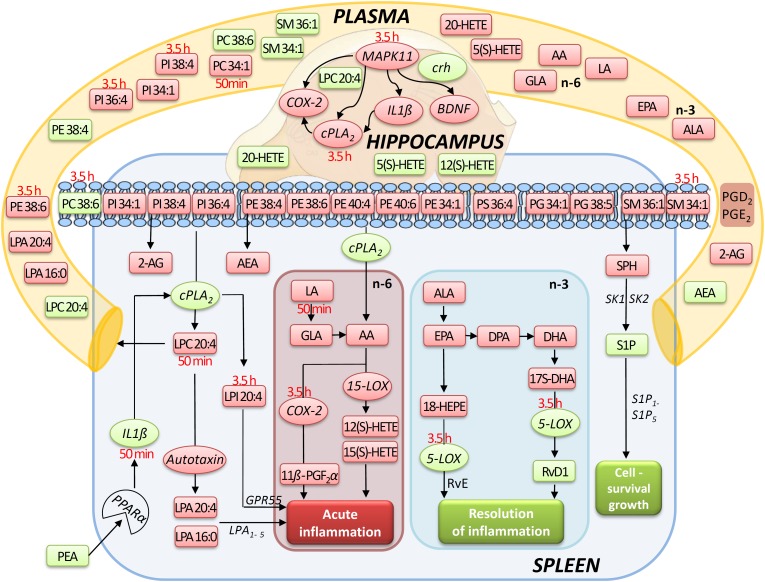
Simplified signaling pathway: this simplified signaling cartoon encompasses all significant molecular changes detected in this study in spleen, hippocampus, and plasma after PEA treatment (PEA/sal). Decreased molecular levels compared to Sal are displayed in red while increased ones in green, respectively. Where lipid level alteration where restricted to a time point, they were specified in red color. The proposed pathways are inferred from published studies that show correlations between the targeted molecules under defined physiological or pathophysiological contexts. This graphic gives also an idea how the PEA-derived splenic molecular phenotype leads to a general state of decreased acute inflammation and increased resolution of inflammation and cell survival/growth. Similarly, possible interrelation between the transcriptional changes (mRNA) by PEA is depicted.

### Lipid Extraction From Plasma

All lipids, except LOX/CYP450/COX-derived ones, were extracted using an adapted LLE method as described in Lerner et al. (2016). Briefly, 1000 μL MTBE/methanol (10:3; v/v), containing the spiking solution (concentrations of internal standards were analog to those used for tissue extraction), as well as 250 μL water containing 5 μM THL/URB597 and 10 μg/mL BHT in final volume were used for lipid extraction out of 40 μL plasma, respectively. Extraction and LC/MRM analysis to target lipids of the LOX/CYP450/COX pathway namely RvD1, HETEs, PGD_2_, 11β-PGF_2_α and PGE_2_ was carried out as previously described, with the exception of using solely negative ion mode analysis (Post et al., 2018).

Except the RNA extraction steps (see RNeasy^®^ Mini Kit protocol), all extraction procedure steps, were carried out at 4°C to minimize *ex vivo* alterations of the endogenous lipid levels.

### Reverse Transcription and Real-Time PCR Analysis

Approximately 120–800 ng isolated RNA per brain sample and 1000 ng per spleen sample were reverse-transcribed in order to generate complementary DNA (cDNA) using the High Capacity cDNA Reverse Transcription Kit with random primer hexamers (Applied Biosystems/Life Technologies, Darmstadt, Germany). The cDNA was diluted 1:10 in H_2_O and amplified in the quantitative PCR (qPCR) using commercial FAM dye-labeled TaqMan assays (Applied Biosystems/Life Technologies, Darmstadt, Germany). The primers used for cDNA detection were specific for the exonic regions of the genes: *Bdnf* (Mm04230607_s1), *PLA_2_* (Mm00447040_m1), *ALOX5* (Mm01182747_m1), *ALOX15* (Mm00507789_m1), *COX-2* (Mm03294838_g1), *PPARα* (Mm00440939_m1), *ALOX12* (Mm00545833_m1), *MAPK11* (Mm00440955_m1), *IL1-β* (Mm00434228_m1), *Enpp2* (Mm00516572_m1), *PPARγ* (Mm00440940_m1). The gene *Crh* (PPM04632A-200) was analyzed using SYBR green primers from QIAGEN instead of TaqMan probes. The reference genes for the TaqMan and SYBR green assays were *GusB* (Mm01197698_m1) and *GAPDH* (F: CTCTGCTCCTCCCTGTTCC/R: TCCCTAGACCCGTACAGTGC), respectively.

The qPCR reactions were performed in duplicates using either TaqMan Gene Expression Mastermix or PowerSYBR Green PCR Mastermix (Applied Biosystems/Life Technologies, Darmstadt, Germany) and analyzed with an ABI 7300 Real-Time PCR cycler (Applied Biosystems/Life Technologies, Darmstadt, Germany).

### LC/MS Qualitative and Quantitative Profiling

Targeted quantitative LC/MRM experiments were carried out with polarity switching using an SCIEX 5500 QTrap triple-quadrupole linear ion trap mass spectrometer (Concord, ON, Canada), as previously described (Lerner et al., 2016, 2018; Post et al., 2018). Via manual tuning, declustering potential, collision cell exit potential, entrance potential, and collision energy of the additionally analyzed lipids S1P, SPH, C1P, PUFAs, and PUFA oxidation products were individually optimized using their calibration standards. The MRM transitions of calibration standards and their corresponding ISTDs, as well as the transitions for the quantification of additionally targeted lipid molecules are depicted in Table 1. For ALA and LA the fragments produced by loss of water exhibited the highest intensity; additional transitions have been used for molecule confirmation. For S1P and C1P, transitions in negative ion mode to their phosphate group were used for quantification, while additional transitions in positive ion mode served as qualifiers. The LC conditions for eCBs and PLs were set as recently described (Bindila and Lutz, 2016; Lerner et al., 2016, 2018). The additional analysis of 7 PUFAs and 2 of their oxidation products 18-HEPE and 17S-DHA was carried out, using the LC conditions for eCB analysis. Chromatographic separation of the sphingosine and ceramide species was carried out together with the PL species. Therefore, the same LC conditions as for PL analysis have been used except of one minor change. To achieve a better ionization, 0.2% formic acid have been added to the mobile phase A, consisting of methanol/water (1:1; v/v) containing 7.5 mM ammonium formate and 0.1% TEA and to the mobile phase B, consisting of methanol/isopropanol (2:8; v/v) containing 7.5 mM ammonium formate and 0.1% TEA, respectively. LOX-, CYP450, and COX-derived lipids were extracted and analyzed using the protocol described in Post et al. (2018), but without the inclusion of eCBs, hence only using the negative ion polarity. MRM transitions and conditions were inferred by manual tuning and are depicted in Table 1.

### Data Processing and Statistical Analysis

Lipids were quantified by Analyst 1.6.2 software (AB SCIEX, Darmstadt, Germany) and MultiQuant 3.0 quantitation package. The obtained values were normalized to the tissue weight/ ml plasma. The analysis of the relative gene expression (RGE) data received from the qPCR was performed using the 2^-ΔΔCT^ method (Livak and Schmittgen, 2001). Target genes were normalized to the reference genes, and the normalized expression levels of the target genes then to that of the control mice. Data were analyzed with GraphPad Prism 4.0 and 8.0 software package (GraphPad Software, San Diego, CA, United States), presented as mean ± SEM and considered significant at a *p*-value < 0.05, e.g., ^∗∗∗^*p* < 0.001, ^∗∗^*p* = 0.001 to 0.01; ^∗^*p* = 0.01 to 0.05. Statistical analyses of the difference between group means were carried out using two-tailed unpaired Student’s *t*-test.

## Results

### Rationale of Study Design, Data Provision, and Biological Matrix Choice

Using the tissue lipidomics and transcriptomic method, as well as plasma lipidomics we investigated molecular effects of sub-chronic, prophylactic PEA administration compared to Sal in both time-dependent manner, e.g., 50 min and 3.5 h post-administration, respectively, and in a treatment-dependent manner, whereby the molecular levels from the PEA and Sal groups at the two time points were summed up and statistically evaluated independent of the time point. We thus, aimed to understand on one hand, a lipid and genomic temporal dynamic upon PEA administration, and nonetheless a cumulative effect of the treatment itself. We chose these time points because of the previously proven molecular effects of PEA (between 50 min and 3.5 h post PEA administration) at an acute symptomatic phase in mice models of epilepsy (Post et al., 2018). This inter-study reference will help elucidate how PEA prepares the body in the face of insults.

Exogenous PEA has been shown to act on both, brain and periphery, and putatively across brain-periphery axis in modulating not only anti-inflammatory but also neuroprotective or symptomatic effects (pain reduction, anticonvulsant) (Mattace Raso et al., 2014; Petrosino and Di Marzo, 2017). The choice of hippocampus was guided by its pivotal function in the on-set of neuroinflammation and neuronal hyperexcitability, which are common features of several neurological diseases (epilepsy, stroke, brain injuries, etc.), and have been shown to be positively affected by PEA (Small et al., 2011; Post et al., 2018). Spleen was chosen as a main organ of the immune system mounting both innate and adaptive immune responses. Splenic immune responses are also modulated in part by a fascinating lipid signaling, for example of the S1P and LPA, involved in immune trafficking and response (Goetzl et al., 2004; Mebius and Kraal, 2005; Bronte and Pittet, 2013). Since, spleen is interposed in the blood stream and bodily’s largest blood filter, we rationalized that administration of a nutraceutical such as PEA would render molecular changes in the spleen, and that PEA’s immunomodulatory properties are partly occurring in spleen. Therefore, PEA’s effect on the splenic lipidome and transcriptome is expected to contribute new aspects of the PEA prophylactic mechanism. Finally, plasma lipidomics can provide both, readout of the PEA’s actions in brain and periphery, and a complementary source to elucidate the PEA’s prophylactic mechanism.

We analyzed changes in the levels of representative mRNAs involved in: (i) inflammation and breakdown of membrane lipids and synthesis of pro- and anti-inflammatory signaling lipids: IL1β, cPLA_2_, COX-2, ALOX 5, ALOX 12, ALOX 15, and autotaxin; (ii) neuronal activity: Bdnf, Crh, and (iii) PEA signaling: PPARα, PPARγ, and MAPK. We have previously showed that a transcriptional over-activation of the Bdnf gene occur specifically in the hippocampal regions of the brain (Lerner et al., 2018) at acute seizure state, underscoring an increased neuronal activity in the onset and progression of seizures (Binder et al., 2001). Hippocampal Crh-expressing neurons have more recently been shown to modulate excitability of the CA3 neurons in response to stress and even affect locomotor activity (Hooper et al., 2018). Due to the reported neuroprotective and anticonvulsant properties of PEA, a transcriptional modulation of Crh and Bdnf upon PEA prophylactic administration was reasoned to be of interest to investigate.

### Spleen

The most significant changes in lipid levels could be detected in the spleen, whereby the great majority of analytes underwent a significant reduction. At 50 min, SPH (^∗∗^), LA (^∗^), ALA (^∗∗^), LPC 20:4 (^∗∗^), SM 34:1 (^∗^), PE 34:1 (^∗∗∗^), PE 38:4 (^∗^), and PE 40:4 (^∗^) were significantly decreased. Thereby, changes of LA and SM 34:1 were significantly stronger as compared to 3.5 h (Figure 2A, 4). At 3.5 h, 2-AG (^∗∗∗^), 18-HEPE (^∗∗^), 17(S)-HDHA (^∗∗∗^), PG 34:1 (^∗^), PG 38:5 (^∗∗∗^), PI 36:4 (^∗∗^), LPI 20:4 (^∗^), PS 36:4 (^∗^), and PE 38:6 (^∗^) underwent significant reduction, while S1P (^∗∗^) and PC 38:6 (^∗^) were significantly enhanced. The LPI 20:4 and PC 38:6 levels were significantly stronger altered at 3.5 h as compared to 50 min (Figure 2B). When pooling both time points, a significant decrease for AA (^∗^), PI 38:4 (^∗^), SM d36:1 (^∗^), PE 40:6 (^∗^) and 11β-PGF_2_α could be detected. The AEA (^∗∗∗^), GLA (^∗∗∗^), EPA (^∗∗∗^), DHA (^∗∗^), DPA (^∗∗^), LPA 16:0 (^∗∗∗^), LPA 20:4 (^∗∗∗^), and PI 34:1 (^∗∗∗^) were significantly decreased at both time points (Figure 2C). A significant downregulation of 5(S)-HETE (^∗∗^), 12(S)-HETE (^∗∗∗^), and 15(S)-HETE (^∗^) was detected with PEA treatment (Figure 5A). Absolute concentrations of RvD1 significantly increased after 3.5 h of PEA treatment as compared to 50 min. Of note, RvD1 was not detected in saline-treated control mice, likely because they are at basal level under limit of detection/quantification or not biosynthesized (Figure 6).

At transcriptomic level after 50 min, only significant changes for IL1-β (^∗∗^) were detected (Figure 7A). At 3.5 h COX-2 (^∗^), ALOX 15 (^∗^), and autotaxin (^∗^) were significantly decreased, while ALOX 5 (^∗^) was significantly increased. The COX-2 and ALOX 5 levels were significantly more altered at 3.5 h compared to 50 min (Figure 7B). Fitting to the general PL breakdown seen in the spleen, mRNA encoding cPLA_2_ was significantly (^∗^) enhanced with the PEA treatment, but not at individual time points (Figure 7C).

### Plasma

At 50 min, AA (^∗∗∗^) and PC 34:1 (^∗^) were significantly decreased, while PE 38:4 (^∗^) was significantly enhanced. PC 34:1 was significantly more decreased as compared to 3.5 h (Figure 3A).

In plasma, more significant changes could be detected at 3.5 h. Two PUFAs; LA (^∗^) and ALA (^∗∗^), as well as four PLs; PI 34:1 (^∗^), PI 36:4 (^∗^), PI 38:4 (^∗∗^), and PE 38:6 (^∗^) showed significantly decreased levels, while four PLs; LPC 20:4 (^∗^), PC 38:6 (^∗^), SM 36:1 (^∗^), and SM 34:1 (^∗∗^) were significantly increased (Figure 3B, 4). The changes in PI 36:4 (^∗^), PI 38:4 (^∗∗^), and PE 38:6 (^∗^) were significantly stronger at 3.5 h as compared to 50 min after PEA injection, thus underlying the time specificity of their significant reduction at 3.5 h (Figure 3B). The eCBs, PUFAs and signaling PLs: AEA (^∗∗^), 2-AG (^∗∗∗^), GLA (^∗∗∗^), EPA (^∗∗∗^), LPA 16:0 (^∗∗∗^), and LPA 20:4 (^∗∗∗^) showed significant changes with treatment and with time. While PEA injection led to enhanced levels of the eCB AEA, all other lipids underwent a significant reduction (Figure 3C). A significant downregulation of 5(S)-HETE (^∗∗∗^) and 20-HETE (^∗∗∗^) was detected with PEA treatment (Figure 5C).

### Hippocampus

The only alteration in hippocampal PL levels was found for LPC 20:4 (^∗^), which was increased 3.5 h after PEA injection (Figure 4). A significant upregulation of the 5(S)-HETE (^∗∗^), 12(S)-HETE (^∗^), and 20-HETE (^∗∗^) was detected with PEA treatment (Figure 5C). PEA administration was previously shown to increase the PEA levels in the brain of control mice (Post et al., 2018) when administered at a dosis of 40 mg/kg, but not at 0.1 mg/kg (Heide et al., 2018).

At transcriptomic level after 50 min, significant changes for IL1-β (^∗^) and Crh (^∗^) were detected (Figure 8A). At 3.5 h after PEA treatment, significant changes for MAPK11 (^∗∗^) and cPLA_2_ (^∗^) were detected, both being significantly stronger as compared to time point 1 (Figure 8B). Treatment-specific significant decrease for COX-2 (^∗^) and Bdnf could be detected (Figure 8C).

## Discussion

Our data evidence that prophylactic sub-chronic PEA administration distinctly effects peripheral immune system and hippocampus, at both transcriptional and lipid molecular level-the exception is COX-2 pathway, which is downregulated in both tissues. Moreover, the downregulation of the COX-2 mRNA is time-point specific (3.5 h post PEA-injection) for both regions. This finding supports the hypothesis of PEA’s action across brain-periphery axis to decrease pro-inflammatory environment via transcriptional modulation of COX-2 pathway. Hence, splenic and hippocampal transcriptional downregulation of COX-2 pathway upon prophylactic PEA administration could balance the hippocampal over-expression of COX-2 upon brain insult (Lerner et al., 2018) and thus prevent development of neuroinflammation (Post et al., 2018). Similarly, PEA’s downregulation of COX-2 pathway in brain and periphery could contribute to building up resistance to bacterial infections and sepsis development (Heide et al., 2018). The transcriptional regulation of splenic and hippocampal cPLA_2_ is divergent (increase in spleen and decrease in HC) and, interestingly, with different consequences on the lipidome in the two regions: in spleen with the exception of PC 38:6, all other phospholipids were downregulated, while, in HC no change of the membrane PLs was detected, except the LPA 20:4 (Figure 9). Even though an upregulation of cPLA_2_ and breakdown of membrane PLs is a hallmark of many diseases, in such cases it is accompanied by the increased production of pro-inflammatory lipids, lipid peroxidation and free fatty acids (Farooqui et al., 2004). This is not the case here in spleen (Figure 2, 5, 9), where it is evident that the transcriptional upregulation of cPLA_2_ is accompanied by a downregulation of COX-2 and 15-LOX with a concurrent upregulation of the 5-LOX mRNA (Figure 7, 9). The increase of resolvin D1 (Figure 6), which is reportedly an anti-inflammatory lipid, along with concurrent decrease of AA and downstream COX and LOX- lipid derivatives (11β-PGF_2_α, 12(S)- and 15(S)-HETE, respectively) (Figure 2C, 5) evidence a shift towards provision of a pro-resolving lipid environment in the spleen via transcriptional activation of the cPLA_2_, 5-LOX, with 5-LOX rendering conversion of omega-3 fatty acids in pro-resolving lipid environment (Figure 9). In line with this mechanism, the omega-6 fatty acids are not used to increase the AA pool (Figure 9) for inflammatory signals, but more likely to remodel/resynthesize splenic membrane PLs, indicated by an increase of PC 38:6 (3.5 h post-injection). The plasma omega 3- and omega-6 fatty acids, as well as PC 38:6 have the same alteration pattern as spleen, which could explain the PEA’s role in positively influencing lipid metabolism in obesity and associated inflammatory consequences (Hoareau et al., 2009).

The downregulation of the splenic pro-inflammatory signaling in favor of pro-resolution signaling is also supported by the transcriptional downregulation of the autotaxin, a major modulator of acute and chronic inflammation (Knowlden and Georas, 2014; Valdés-Rives and González-Arenas, 2017), and by the subsequent decrease of its extracellularly produced LPA lipids (LPA 20:4 and C16:0, respectively) (Figure 2C, 7B). LPA is one of the main signaling lipids in spleen that is multi-functional in modulating immune response (Saba, 2004). Recently, autotaxin-LPA pathway has been implicated as a main player in aberrant immune responses and development of inflammatory responses. The PEA’s effect on this pathway is therefore supporting its role as a positive immunomodulatory and warrants further investigation. PEA prophylactic administration modulates (increases) the level of S1P which is a vital lipid signal in the splenic immune function. The increase of S1P likely occurs through the breakdown of SM 36:1 (Figure 9) and conversion through SPH to S1P. S1P, via binding to its S1P1R receptors, is the bioactive lipid regulator of both innate and adaptive immune system and serves a plethora of biological functions in the splenic immune system. From our data it is impossible to reveal the role of PEA-derived S1P increase. However, considering the reported immunomodulatory properties of PEA it is pertinent to conclude that PEA-derived S1P increase must be part of a beneficial immune system boosting, which certainly needs further investigation. A time-specific (50 min) increase of IL1-β was also detected in spleen. Even though this molecule is mainly described as a pro-inflammatory cytokine that activates the cPLA_2_ leading to pro-inflammatory cascades, it is obvious that this was not the case in spleen. In contrast, IL1-β and cPLA_2_ activation is accompanied by a shift in signaling towards decreasing inflammatory cascade and boosting pro-resolving lipid environment (Figure 6, 9). This is also supported by the fact that IL1-β increase is terminated after 50 min. It is therefore, more likely that (short) activation of IL1-β boosts in this case immune defense and functions as an immunoadjuvant upon PEA prophylaxis (Dinarello, 1998). In contrast, in HC mRNA levels of both, IL1-β and cPLA_2_ are downregulated along with those of MPAK11 and COX-2. These indicate a distinct regulation by PEA of neuroinflammatory pathway in HC than inflammatory pathway in spleen. The concerted decrease of these molecules in hippocampus point towards a MAPK11-induced downregulation of cytokine production and cytosolic cPLA2 with a downstream regulation of COX-2 as well (Wang et al., 2008). Nevertheless, this broad downregulation of the hippocampal pro-inflammatory tone explains the reported anti neuro-inflammatory properties of the PEA in various brain injuries, recently demonstrated for multiple sclerosis as well (Orefice et al., 2016). LPC 20:4 has reportedly been implicated in triggering inflammation via activation of COX-2 (Brkić et al., 2012). Because in hippocampus, the mRNA of the COX-2 is down-regulated, but LPC 20:4 up-regulated, modulation of hippocampal COX-2 is independent of LPC 20:4. It follows then that MAPK- induced cPLA_2_/ IL1-β /COX-2 pathway is the more likely venue for PEA modulation of hippocampal inflammation. What is the source and function of hippocampal upregulation of LPC 20:4 upon PEA administration is not apparent from our data and remains to be determined. Similarly, increase and function of 5(S)-, 12(S)-, and 20- HETE in HC remains an open question to be investigated, especially, since none of the investigated LOX- mRNAs are altered, and provision of more data on CYP450 pathway is required. Indeed, in our study neither the translation of genes nor enzymatic activities were investigated which would answer and guide the interpretation of such lipid changes, e.g., hippocampal increase of LPC 20:4 and 5(S)-, 12(S)- and 20- HETE and/or plasma increase of PE 38:4, PC 38:6, LPC 20:4, SM 34:1, and SM 36:1.

Of general note is that the biochemical processes leading to the significant changes of the targeted lipidome upon PEA administration are time-wise diverse, as evident from the time-resolution lipid analysis. A broader lipidome change occurs at 3.5 h than at 50 min post-administration (Figure 2–4). We, therefore consider that generally, investigating a temporal dynamic of lipidome is advantageous to exhaustively reveal lipids involved in a particular biological context such as here described.

A particular feature of prophylactic PEA effect in the HC is the transcriptional downregulation of Bdnf gene and upregulation of Crh gene. Bdnf-mRNA downregulation by PEA could counterbalance aberrant increase elicited by brain insults such as the case in epilepsy (Lerner et al., 2018), hence downregulating the underlying increased neuronal activity rendering decreased seizure intensity (Post et al., 2018). More recently, a hippocampal neuronal subpopulation was shown to express Crh which modulates hippocampal excitability and maintain adaptive network excitability. Inhibition of Crh-expressing neurons increased locomotor activity while selective ablation of Crh-neurons in HC led to increased seizure susceptibility (Hooper et al., 2018). In view of this line of evidence, the transcriptional upregulation of hippocampal Crh gene upon PEA prophylactic administration is really interesting, and opens new venue of research to explain anticonvulsant effects of PEA administration. Collectively, hippocampal decreased neuroinflammatory pathways MAPK11/IL1-β/cPLA_2_/COX-2 and decreased hippocampal excitability as indicated by decreased Bdnf mRNA along with increased Crh mRNA expression could contribute to the neuroprotective, anti-neuroinflammatory, and anticonvulsant properties described for PEA in epilepsy and other brain insults. A transcriptional activation of PPARα gene by PEA at the time points investigated here in control mice was not detected, which certainly does not exclude its activation.

## Conclusion

Prophylactic PEA administration generated a complex molecular phenotype at transcriptomic and lipid level in spleen and brain and blood. Because, the PEA prophylaxis is reportedly effective it can be concluded that the induced molecular phenotype is beneficial to the body in facing various negative insults (infections, neuro injuries, etc.).

Finally, the resulting new insight into molecular plasticity is a step forward in understanding what can be targeted by PEA in prospective preventive care measures and hence help guide prophylactic or adjuvant approaches for example for groups at high risk of inflammation (through bacterial exposure) or neuro injury (epilepsy, stroke brain injuries, etc.). Concurrently, our findings open new questions into the multi-facetted mechanisms of PEA and new inroads of research into the prophylactic effects of PEA on immune system and central nervous system.

Future studies focusing on gender and age-dependent response to PEA administration should clarify mechanistic aspects of PEA and its applicability in general health care. Of note is that the methodology presented here is of general applicability in studying nutrition and nutraceutical effects on tissue molecular composition and consequential affected pathways.

The circulatory lipid profile reflects major molecular events in the brain and peripheral immune system upon PEA administration, so that plasma lipidomics can be a promising clinical tool to monitor and possibly predict response to nutraceutical/nutrition based therapy and prophylaxis.

## Ethics Statement

Experiments were performed according to the European Community’s Council Directive of 22 September 2010 (2010/63EU) and approved by the local animal care committee of the German Rhineland-Palatinate (file reference: 23 177-07/G16- 1-075).

## Author Contributions

RL carried out the main lipidomic experiments, prepared, interpreted and processed the data, contributed to manuscript writing. DPC performed the transcriptomic experiments and processing of the mRNA data. JMP performed the animal experiments and part of the lipidomic experiments. BL contributed to data interpretation. LB coordinated the study contributed to data interpretation and manuscript writing.

## Conflict of Interest Statement

The authors declare that the research was conducted in the absence of any commercial or financial relationships that could be construed as a potential conflict of interest.

## Abbreviations

2-AG: 2-arachidonyl glycerol
AA: arachidonic acid
ACN: acetonitrile
AEA: arachidonoyl ethanolamide
ALA: alpha-linolenic acid
BBB: blood–brain barrier
Bdnf: brain-derived neurotrophic factor
BHT: butylhydroxytoluene
C1P: ceramide-1-phosphate
CB1R: cannabinoid receptor type 1
CER: ceramide
COX: cyclooxygenase
Crh: corticotropin releasing hormone
DHA: docosahexaenoic acid
DPA: docosapentaenoic acid
eCBs: endocannabinoids
eiCs: eicosanoids
EPA: eicosapentaenoic acid
FAAH: fatty acid amide hydrolase
GLA: gamma-linolenic acid
HC: hippocampus
HETE: hydroxyeicosatetraenoic acid
ip: intraperitoneal
ISTD: internal standard
LA: linoleic acid
LLE: liquid–liquid extraction
LPA: lysophosphatidic acid
LPC: lysophosphatidylcholine
MAPK: mitogen-activated protein kinase
MRM: multiple reaction monitoring
MTBE: methyl *tert*-butyl ether
NAAA: *N*-acylethanolamine acid amidase
NAPE-PLD: *N*-acyl phosphatidylethanolamine phospholipase D
PA: phosphatidic acid
PC: phosphatidylcholine
PE: phosphatidylethanolamine
PEA: palmitoyl ethanolamide
PG: phosphatidylglycerol
PGD_2_: 11β-PGF_2_α, 11beta-prostaglandin F2alpha, prostaglandin D_2_
PGE_2_: prostaglandin E_2_
PI: phosphatidylinositol
PL: phospholipid
cPLA_2_: cytosolic phospholipase A_2_
PPAR: peroxisome proliferator-activated receptor
PS: phosphatidylserine
PUFAs: polyunsaturated fatty acids
RvD1: resolvin D1
sal: saline
S1P: sphingosine-1-phosphate
SM: sphingomyelin
SPH: sphingosine
TEA: triethylamine
URB597: KDS-4103, 3′-(aminocarbonyl) [1, 1′-biphenyl]-3-yl –cyclohexylcarbamate
